# A Review of the Relations between the State and the Medical Profession in New York1Introductory address to the forty-ninth course of lectures, Medical Department, University of Buffalo, September 24, 1894.

**Published:** 1894-11

**Authors:** Frank Whitehill Hinkel


Buffalo Medical and Surgical Journal

VOL. XXXIV. NOVEMBER, 1894. NO. 4.

### Original Addresses.

### A REVIEW OF THE RELATIONS BETWEEN THE STATE AND THE MEDICAL PROFESSION IN NEW YORK.[1]

BY FRANK WHITEHILL HINKEL, A. M., M. D.

WHEN asked a few days ago to welcome you tonight, to the forty-ninth term of medical lectures held in this university, with a brief address on some subject cognate to your purpose in assembling here, I recalled that this pleasant duty was allotted to me eight years ago.

The assemblage at that time was held in the lower of the two bare and rather dingy lecture-rooms of the old university building at the corner of Main and Virginia streets. The recollection of that occasion, though it is so recent, affords points of sharp contrast between then and now that have suggested the theme of my address tonight. It is not the material changes in our school that occur to me with force, nor that I wish to impress upon you. This spacious, well-lighted, well-ventilated amphitheater, the well-equipped laboratories, this imposing building, excellent in its appointments and architectural effect—these are, indeed, in striking contrast to the surroundings in which I then addressed the incoming classes. I would not underrate the importance of these improvements, nor the benefits that innure to you from the increased opportunities and facilities they afford ; but it is the significance of this costly building and equipments, and the increased requirements and obligations that have devolved upon you therewith, that I wish to call to your attention.

Great achievements, like great trees, have widespread roots and result from long and slow-acting forces. This building, in its

1. Introductory address to the forty-ninth course of lectures, Medical Department, University of Buffalo, September 24, 1894.

conception, planning and completion, has a history of wide ramifications and no mean interest. The history of that which it typifies and which alone gives it significance—the history of the increasing importance of medicine in its relations to the state, and of the increasing qualifications and protection that the community is demanding of us,—this is, indeed, deep-rooted in the past and complex in its relations.

I shall endeavor to present to you, with such brevity as the occasion demands, a review of the relations of the medical profession to the people of this state, as exemplified by the various legislative enactments providing for the licensing of physicians, their qualifications, etc. I have chosen to review the medical history of this state as of most interest and practical instruction to us, and because it is in this state that the relations between the state and medical profession have reached the highest plane yet achieved in this country, both as regards the well-being of the state and the dignity and honor of the profession.

The first act to regulate the practice of medicine passed in this state was on March 23, 1797. By it the candidate for license was required to show that he had studied medicine for a term of four years with a "respectable physician or surgeon." A sworn certificate to this effect from his preceptor was presented before either the governor, the chancellor of the university, the judges of the Supreme Court or the attorney-general, who endorsed upon it a permit to practise. This was then filed in the clerk's office. The degree of M. B. or M. D. from a medical institution qualified to practise on filing of the diploma. There were then in the United States but three medical schools, the University of Pennsylvania, Columbia College and Harvard. You will note that graduation from a medical school was not essential to the obtaining of a license, nor was there any examination as to the candidate's fitness, and that the licensing power was solely in the hands of the legal profession. This subordination of the medical to the legal profession was in accord with colonial usage.

In 1806, a new act altered radically the relations of medicine to the state and greatly increased both its importance and its responsibilities. By this act the physicians "now authorized to practise" were directed to assemble at a certain date in the several counties of the state and form county medical societies. Each county society was to appoint a board of censors, whose duty it was to examine all candidates presenting themselves for license to practise. These several boards of censors were constituted the sole powers from which licenses to practise could be obtained. Three years of medical study were required before the candidate was admitted to examination. Each county society was required to appoint delegates to meet annually at Albany, these delegates to constitute the Medical Society of the State of New York.

This bill has two striking features of great import to medicine. For the first time it entrusted the control of medical practice solely to the medical profession,—not to magistrates and lawyers. Practitioners of medicine were constituted, as it were, a branch of the legislature. A state society, representative of medicine, met annually at Albany to supervise medical education and medical practice in the state, and to lay before the legislature and the people whatever concerned the well-being of the state from the medical standpoint! It was an honorable and a responsible obligation laid upon the members of our profession, and it appears that while it rested upon them it was honorably and responsibly fulfilled in the main. The annual addresses of the successive presidents for many years after the foundation of the state society portray a keen consciousness of the important relations the society bore to the state, and of the interest the legislature and the people took, or were supposed to take, in its proceedings.

The other feature of this bill of abiding interest, is that it provides for an examination of the student as to fitness, by representatives of all the practitioners of medicine in the state, before he was allowed to become a member of the profession. For, by this bill, the degree of M. D. obtained from a medical school did not license to practise without the censors' examination. Four years' of study with a preceptor were required of the candidate, but the degree of M. D. from a medical school was not an essential prerequisite to examination.

By these two features of this enactment the medical profession in this state was raised to the honorable position of a self-regulating, highly responsible, peculiarly privileged organization, having delegated to it functions of grave moment to the community.

This relation of the profession with the state continued in force, in its important details, for many years. There were two weak features in this scheme of medical control, and it is to the credit of the profession that it was well aware of them and sought to correct them. The report of a select committee of the Medical Society of the State of New York on the subject of medical education, presented at the meeting of 1840, points out these defects with much clearness and force. This committee was the outcome of agitation in several previous years, and it made an exhaustive report. This report laments the inadequacy of the education required preliminary to the beginning of medical study. It points out the great advantage to the profession and to the individual practitioner of sufficient preliminary mental discipline, but it has no adequate suggestion for the enforcement of such qualifications. The other and very palpable weakness in the law lay in the multiplicity of licensing bodies with varying standards of requirements. The report asserts that in 1840 there were "at least fifty bodies of men in the state authorized to permit an individual to practise physic and surgery." The only remedy suggested was to lessen the number of licensing bodies to correspond to the senatorial districts, and at the same time to increase the size of each.

In 1835, an act was passed by the legislature incorporating the medical faculty of Geneva College as a medical school, and its trustees were empowered to confer the degree of M. D. This degree was declared a license to practise medicine in this state. This was the first clip of the shears to weaken our medical Samson and take away his power and his responsibility. By this enactment the power to license and regulate medical practice was taken from the profession at large, through its delegates, and placed in the hands of a private corporation—and that a corporation tempted withal, by every incentive, to increase the number of licentiates. By an earlier enactment the board of regents of the University of the State of New York was enabled to confer the degree of M. D., which degree was a license to practise. This applied practically to the graduates of the College of Physicians and Surgeons in New York—the medical school of the State University. In the hands of the regents such power was safe. When it was further extended, its tendency was harmful and prejudicial to the interests of the State and the medical profession, endangering the one, degrading the other.

In 1837, the University of the City of New York was authorized to establish a medical faculty whose degrees were a license to practise. In 1839, the Albany Medical School was granted like permission. With these exceptions the control of medicine remained in our hands until 1844, when the legislature enacted a law that took away our high prerogative, and created in effect a system of free medicine in this state. It removed all restrictions from the practice of medicine and put unlicensed practitioners on exactly the same basis with those who were licensed. In this slough the state and the profession wallowed until 1872. The slime of it still adheres to us.

The causes that led the state to reverse its policy of nearly forty years, and to revoke the responsible duties heretofore delegated to our profession, I have not had time to investigate with that care their interest and importance warrant. By inference, I deduce that they were quite complex. The annual addresses of the presidents of the state society, and the reports of certain of its committees, for a number of years preceding the repeal of the medical practice bills of 1806 and subsequent years, contain more or less extended reference to the attempts of various "isms" and "pathies" to obtain entrance to the medical fold other than by the appointed gates. The mistaken, if often well-meant and unselfish, zeal which led to injudicious assaults upon the budding "fad" of homeopathy and occasionally to inexcusable treatment of its individual adherents, was a not unimportant factor, I believe, in producing a "free-medicine" bill. We may deduce a moral, in passing, that undignified invective and ecclesiastic-like intolerance cannot but weaken the best of causes before a public tribunal. The "under dog" gets the sympathy if there be manifest disparity in size and strength.

About this time, commensurate with the rapid extension of our country and its great material development, began the establishment of medical colleges in various thriving towns throughout the United States. Helpless New York must have been a rare market for the raw products of many of these schools. Without efficient supervision as to the character and extent of their teachings, without the former valuable separation (in this state at least) of the graduating from the licensing power, with every incentive that greed or ambition could give to graduate the largest number possible, the wonder is that these schools did not more abuse their absolute power.

Unquestionably, the *personnel*, the character and the dignity of the medical profession were seriously lowered by this combination of circumstances, not only in this State but throughout the United States. And yet we must remember that this clipping of the prerogatives of our profession, this lowering of its plane, was one manifestation of the spread of the democratic idea that has affected everything in our social, political and even religious life. Our medical schools but fulfilled the "law of supply and demand," as they multiplied practitioners with such facile rapidity. New communities ever springing up needed medical care, and he who knew a little was more serviceable in many ways, and no more dangerous in some ways, than he who knew nothing. Our great civil war created an enormous demand for military surgeons, many of whom, it must be admitted, were as raw recruits as—and a deal more dangerous than—the "boys in blue" or grey, as the case may be, upon whom at first they operated. Higher standards and more rigorous requirements would have found few outside of our older communities who were equal to them in mental training. For, despite the "free school" system throughout our country, higher education, until very recently, has in no wise kept pace with our commercial and material development.

During these "dark ages" of the profession in this state and at large, there was no lack of shining lights, it is true, who, by personal example and teaching, kept high ideals and aims before our young men. The records of the meetings of the state society continuously contain addresses and committee reports deploring the existing relations of the medical profession and the community and urging various reforms. Two medical schools stand out illustrious in this time for their efforts in behalf of a higher medical education. The Medical School of Harvard University, early in the '70's, adopted a graded course of instruction, and required three courses of study of eight or nine months each before graduation. Certain educational requirements were held necessary for admission to study in the school. The University of Pennsylvania followed closely with the same requirements—a proud record for the alumni of these institutions.

That I have not shaded the history of this time too dark; that I have not laid too large a share of responsibility on the medical schools in asserting that the power of granting license to practise in addition to the medical degree, permitted by law at this time, was detrimental to their standards and injurious to the state,—let me present some statements from the fifteenth annual report of the Illinois State Board of Health. I wish to acknowledge, in passing, though time forbids interesting details, the powerful influence for better medical education exerted by the acts and reports of this board since 1880.

In 1840, there were seventeen medical schools in the United States, of which two were in this state. In so recent a year as 1880, fifty-seven of the schools in existence in the United States furnished the following educational data: Forty-three were regular; ten were homeopathic; three were eclectic; and one, of a nondescript kind, was classed as "miscellaneous." Of these fifty-seven schools only fifteen—fourteen regular and one homeopathic—exacted any qualifications of their matriculates. The other forty-two made no inquiry of their fitness for entry upon the study of medicine. Forty-one of the fifty-seven schools conferred the degree of M. D. after attendance upon two annual courses of lectures of an average duration each of but 22.6 weeks—scant five months. Only thirteen regular and two homeopathic schools required three annual courses of lectures. More than half the regular schools graduated upon two annual lecture courses averaging less than twenty weeks each. In only fifteen schools were the lecture courses graded. Such a showing for only fourteen years ago!

But light began to break upon the "dark age" in this state in 1872, when the efforts of the profession and the influence of enlightened public opinion secured legislation looking to the restraint and control of medical practice. In 1874, in 1880 and again in 1881, various laws were enacted for the same object. It is unnecessary to detail them. They proved unsatisfactory in results, for they were somewhat obscure and contradictory in terms, and they had in common the fatal defects that they did not restore to the medical profession the selection of and responsibility for its membership, and did not sever the licensing from the degree-conferring power. But earnest and persevering members of our profession renewed the struggle in the past decade, and at last the ancient landmarks have been restored—and a good deal bettered in the restoration. When we consider their recent success in securing the enactment of the medical laws under which we meet this evening, it is a matter of pleasure and pride to recall that this "reform bill," fraught with so much of promise and benefit to our profession, had its inception here in Buffalo. Probably no one man did more active or efficient service in procuring the enactment of the present law than a member of our faculty, Professor H. R. Hopkins.

I need not repeat the details of this law to you, so recently beginning the study of medicine or so soon to enter practice under its auspices. I would call your attention briefly to the relations it establishes anew between our profession and the state and to the advantages and the obligations it entails.

1. As of yore, the state delegates to the medical profession the sole power to decide the fitness of candidates for entry to its membership. The examining board, that decides the fitness of every candidate by examination, is appointed by the Regents from nominees from the official medical societies representing the entire medical profession of the state. Hence, the licensing power lies ultimately directly with the medical profession. Two points of superiority over the old laws of 1806 to 1844 merit our attention in this connection. One, that the degree of M. D. is a prerequisite to examination for license. This degree must be obtained from a medical school having a three years' course and registered by the regents of the University of the State of New York as having a satisfactory course of study. The other, of peculiar interest, is the constitutional oath of office required of each examiner, such as is required of any civil officer of the state. The medical profession, through this delegate from its ranks, becomes a part of the executive machinery of the state. I need lay no stress on the honor and the obligation this lays upon us.

2. The license examinations are entirely separate from the examinations for the degree of M. D. which must precede them. By the system employed in the state examinations the examiner does not know the name of the applicant whose paper he examines, nor what school has conferred upon him his degree of M. D. The personal equation is reduced to its lowest quantity.

3. A certain minimum of preliminary education is required of the student desiring to matriculate in a medical school in this state. This is enforced by requiring regents' certificates of proficiency from every matriculate, without which he is not admitted to the license examinations. Thus a defect in the old law, commented on before, is obviated in part.

4. Of inexpressible value is the uniformity of standard obtained in the examinations by this law. You will remember the grave defect in the old law by which there were as many standards as there were county societies. This is remedied effectually through the regents of the University of the State of New York.

No institution in this state is more creditable to it than the University of the State of New York; no office in its gift is more honorable and important than that of regent of this university; yet I feel safe in asserting that no institution and no offices of this state are so little known and understood by intelligent citizens as these. Some, at least, of my hearers have passed "regents' examinations." How many of you know just who the regents are, or why the examinations are so called? George William Curtis, in his address as chancellor of the University of the State of New York before the University Convocation in 1890, recounts, amongst other anecdotes to the same effect, that "one of the most eminent lawyers of the state" begged to congratulate him a few months before on his election as chancellor, and at the same time asked "where the university was situated." Supported by this anecdote, and more by recent observations of my own, I believe a few words about the regents are necessary to a clear understanding of the merits of the medical law of '93, though I have already transgressed on your time and patience.

In 1787, the legislature, under the influence of the wonderful state-craft of the illustrious Alexander Hamilton, created the University of the State of New York, substantially as it now is. Its powers were enlarged and confirmed on the original lines in 1889. These powers are vested in nineteen regents elected for life by the legislature with the same formality as that attending the election of the United States senators. It is the only life office in the state. It is thus raised above party politics, and the regents' chairs have been, and are, filled by honorable and cultured men. Buffalo has the honor of having one regent, Mr. T. Guilford Smith. The Governor, Lieutenant-Governor, Secretary of State and the Superintendent of Public Instruction are *ex-officio* members of the Board of Regents. The functions of the university are the control and supervision of the academies, high schools, colleges, universities, professional and technical schools—in short, all the academic and higher education of the state, including the libraries and museums and the legal and medical license examinations. The primary education of the state is under the control of the superintendent of public instruction; but by controlling the entrance examinations of the high schools the regents set the standard for the grammar schools. The regents have large powers and they can alter or revoke for cause the charters of the various educational institutions under their control. These number, at present, between 500 and 600. The University of the State of New York is a supervisory and administrative, not a teaching, institution. It has no buildings and no classes. Its purpose, as defined by law and fulfilled by the regents, is to encourage and promote education in advance of the common elementary branches. This it accomplishes by the standards set in its examinations in our high schools, by the distribution of an academic fund, by inspectors, by convocations of our leading teachers and by its constant subtle stimulus to higher educational aims and better results.

We have here a mechanism possessed by no other state for securing a high, uniform and fair system of examinations; and the framers of our bill wisely entrusted this important function to the regents, with results obtainable in no other way. The regents are able to enforce uniform matriculation requirements as could no other power. They are able, by means, time fails me to detail, to render uniform the examinations for license by the boards of the different "schools" of medicine and they will surely carry the standard higher and higher, instead of allowing it to lapse with time as might occur with any other supervising institution. By their registration of the medical schools that demand satisfactory courses of study before conferring their degrees and by admitting to examination for license only the graduates of these schools, the regents exert an immense influence toward higher standards in the medical schools of the entire country.

Under the present regulations our profession now stands, in relation to the state, in the proud and responsible attitude of the accredited guardians of the public health and safety as pertains to medical matters. We are provided with machinery to enforce, in this state, a fair degree of preparation for medical study, a sufficient course of study in quantity and quality in the medical schools, whose graduates wish to practise in this state, and fairness and uniformity in the requisites for license—for admission to fellowship in a body again raised to unusual honor in the state. Let us remember *noblesse oblige*. It will some day be your duty, through the county and state societies, to guard carefully the charge thus entrusted you, as well as in your private and professional life.

Do not look upon the license examinations as a burden laid upon you. Heed not the low-minded suggestion that it is a manifestation of "trades-unionism." You have seen that it has improved your schools and has raised, and will still further raise, your future profession in the estimation of your fellow-men,—and, of yet greater importance, in your own estimation. Personally, it will be a matter of pride to hold a license to practise from the State of New York. It will give you standing in the profession wherever you go, as has heretofore been the case solely with the alumni of a very few of our medical schools in this country. The report of the examination department of the University of the State of New York for February, 1894, says: "No other state examining board has yet been registered as maintaining a standard equal to that fixed by the New York law." This report says, furthermore: "The greatest gain from the new system is in its deterring many incompetent men from becoming candidates; but of this there can never be statistics. . . . Formerly, without considering those from other states who came to New York, between 600 and 800 physicians were annually graduated by New York medical schools, with authority to practise medicine in the state. Under the present law, the total number licensed annually will, probably, fall short of 400. The new law, while an effective barrier to the ingress of the incompetent, has also operated to raise the standard of preliminary education and to improve the tomes of study and methods of teaching in the medical schools."

If you pursue your studies faithfully and with due diligence, after you receive your degree from our school you need have no fear of the licensing board. The medical profession, unselfish and high-minded in its purpose, will welcome you to its ranks. It seeks not to embrace only the brilliant or the talented; it aims only to exclude the positively incompetent or morally unfit. With this assurance and incentive to win membership in a profession that today stands higher in the state and the community than ever before, and with greater possibilities and responsibilities upon it, I welcome you cordially to the ensuing course of lectures in the Medical Department of the University of Buffalo.

---

VAGINAL INJECTIONS AFTER LABOR.—Eberhart maintains that injections are always needed after delivery when there is gonorrhea, when there is any other profuse discharge, when the vaginal mucus is fetid, when the temperature rises, and when any obstetric operation has been performed. Otherwise the injections are not needed in normal labors in private practice. In hospitals they must always be used. Eberhart has seen the best results follow preliminary vaginal douches, after Kaltenbach's practice. He uses them in private as well as in hospital. He has discarded sublimate, and employs a one per cent. lysol solution. For intrauterine injections lysol should always be employed.—*Centralbl. f. Gynäk.—University Med. Mag.*, September, 1894.

